# Surface Hydrophobic Modification of Fifth-Generation Hydroxyl-Terminated Poly(amidoamine) Dendrimers and Its Effect on Biocompatibility and Rheology

**DOI:** 10.3390/ma2030883

**Published:** 2009-08-04

**Authors:** Paul D. Hamilton, Donghui Z. Jacobs, Brian Rapp, Nathan Ravi

**Affiliations:** 1 Research Branch, Department of Veterans Affairs Medical Center JC 151, 915 N. Grand Blvd. St. Louis, MO 63106, USA; E-Mail: Hamilton@vision.wustl.edu (P.H.); 2 Department of Ophthalmology and Visual Sciences, Washington University School of Medicine, Box 8096, 660 S. Euclid, St. Louis, MO 63110, USA; E-Mails: zhanggloria@hotmail.com (D.J.); rappb1@gmail.com (B.R.); 3 Department of Energy, Environmental and Chemical Engineering, Washington University in St. Louis, MO 63130, USA; 4 Executive Branch, Department of Veterans Affairs Medical Center JC 151, 915 N. Grand Blvd. St. Louis, MO 63106, USA

**Keywords:** dendrimers, surface modification, amphiphilic molecules, biocompatibility, rheology, lens crystallins

## Abstract

Water-soluble, commercially-available poly(amidoamine) (PAMAM) dendrimers are highly-branched, well-defined, monodisperse macromolecules having an ethylenediamine core and varying surface functional groups. Dendrimers are being employed in an increasing number of biomedical applications. In this study, commercially obtained generation 5 hydroxyl-terminated (G_5_OH) PAMAM dendrimers were studied as potential proteomimetics for ophthalmic uses. To this end, the surface of G_5_OH PAMAM dendrimers were hydrophobically modified with varying amounts of dodecyl moieties, (flexible long aliphatic chains), or cholesteryl moieties (rigid lipid found in abundance in biological systems). Dendrimers were characterized by ^1^H-NMR, DLS, DSC and HPLC. The hydrophobic modification caused aggregation and molecular interactions between dendrimers that is absent in unmodified dendrimers. *In vitro* tissue culture showed that increasing the amount of dodecyl modification gave a proportional increase in toxicity of the dendrimers, while with increasing cholesteryl modification there was no corresponding increase in toxicity. Storage and loss modulus were measured for selected formulations. The hydrophobic modification caused an increase in loss modulus, while the effect on storage modulus was more complex. Rheological properties of the dendrimer solutions were comparable to those of porcine lens crystallins.

## 1. Introduction 

Water-soluble, commercially-available poly(amidoamine) (PAMAM) dendrimers are highly branched, well defined, monodisperse macromolecules having an ethylenediamine core and surface functional groups of one of three main types (primary amine, hydroxyl, or carboxylate termini) [1,2]. These dendrimers are easily functionalized and have shown potential applications in nanomedicine such as drug carriers, gene delivery vectors, biosensors, imaging or contrast agents, cell labeling, bioartificial liver systems and tissue scaffolds [3,4,5,6,7,8,9,10,11,12,13,14,15,16,17,18,19]. Such promise in biological uses has aroused great interest in studies on the biocompatibility of PAMAM dendrimers and their derivatives. Since one of the earliest systematic investigations on the toxicity, immunogenicity, and biodistribution of poly(amidoamine) dendrimers by Roberts *et al.* in 1996 [20], research in the area has grown exponentially. In addition to *in vitro* cytotoxicity tests using a wide variety of cell lines, a number of biological properties have been tested *in vivo*, and toxicity (cyto-, hemato-, or myo-), immunogenicity, biodistribution, intracellular fate, etc, have been explored [3,7,8,9,10,11,20,21,22,23,24,25,26,27,28,29]. One of the primary findings in these studies on poly(amidoamine) dendrimers is that the nature of their peripheries, in terms of charge and surface functionality, is of importance in determining the biological performance of PAMAM dendrimers. For example, it was found that amine-terminated PAMAM dendrimers with positive charges on the surface at physiological pH were more toxic than dendrimers having carboxylate termini [26]. It was proposed that the higher toxicity might be due to the cationic amines which would tend to bind to negatively charged cell membranes. The toxicity of amine-terminated dendrimers, however, can be greatly reduced by surface modification with chemically inert molecules such as fatty acids, phosphorylcholine, acetyl groups or polyethylene glycol [4,7,23,24,27,30]. On the other hand, coupling of hydrophobic flurophores to cationic poly(amidoamine) dendrimers enhanced the capabilities of the dendrimers to disrupt endosomal membranes, improving their delivery characteristics for nucleic acids [31,32]. Moreover, since the biological properties of poly(amidoamine) dendrimers are tuned with variations of their terminal functionality, it is important to qualify the biocompatibility of any new classes of derivatives of PAMAM dendrimers proposed for biological applications.

Our intended biological purpose for PAMAM dendrimers is the possibility of using them as replacements or mimics of the lens crystallin proteins. Presently, lens replacement with cataract surgery is performed using a prefabricated intraocular lens that is injected into the lens capsular bag. It is our goal to develop a prosthesis that would mimic the original biological lens that could be injected into and fill the lens capsular bag, forming a naturally auto-focusing intra-ocular lens. To accomplish this, we are developing nanocomposites made of two classes of molecules, one to mimic the insoluble structural proteins of the lens, and the other to mimic the soluble or globular crystallin proteins of the lens [9,33,34,35]. The structural proteins supply the majority of the elastic properties of the lens while the crystallin proteins are responsible for giving the lens the majority of its viscous and optical properties, which include a high refractive index. Crystallins have multiple intermolecular interactions and form a complex mixture that behaves as a non-Newtonian viscoelastic liquid [36]. We are exploring the possibility of PAMAM dendrimers as building blocks and hydrophobic molecules as stickers to make up lenticular proteomimetics to substitute for lens crystallin protein mixtures. Upon testing the rheological properties of various dendrimers, we found that at similar concentrations with matching refractive index and density, the G_5_OH dendrimer approximated the unfractionated total lens porcine crystallin mix at physiological concentrations in certain key parameters such as storage and loss modulus [37]. For the purpose of further understanding the tunable biological profile of PAMAM dendrimers by adjusting their peripheral nature, we partially conjugated G_5_OH PAMAM dendrimers with two kinds of hydrophobic molecules, namely dodecane and cholesterol. The synthesis and aggregation properties of the G_5_OH PAMAM dendrimer modified with 2% cholesteryl groups was reported previously [38,39]. In this paper, we report the results of the *in vitro* cytotoxicity of these amphiphilic dendrimers toward cells in tissue culture, using Chinese Hamster Ovarian (CHO) cells and Pig Lens Epithelial (PLE) cells isolated in our lab. We have also included certain pertinent physical and rheological characterizations that have not been previously reported. 

## 2. Results and Discussion

Our primary interest in dendrimers is their use as proteomimetics, and specifically as substitutes for lens crystallin proteins [33,34]. While many studies on PAMAM dendrimers often focus on ionic (i.e. amine or carboxyl terminated) dendrimers because of their potential uses in controlled drug or gene delivery, we selected G_5_OH (hydroxyl-terminated) PAMAM dendrimers to study the effect of hydrophobicity on their biological behaviors as they are more suited to our intended ophthalmic applications. In general, dendrimer generation number accounts for the size of the dendrimer while surface charge and functionality are two major factors which influence the properties of dendrimers. If the effect of charge is negated, the influence of surface functionality, that is, hydrophobic effects in our case, would dominate the responses of the dendrimers. 

### 2.1. Dendrimer Modification and Characterization

**Figure 1:**
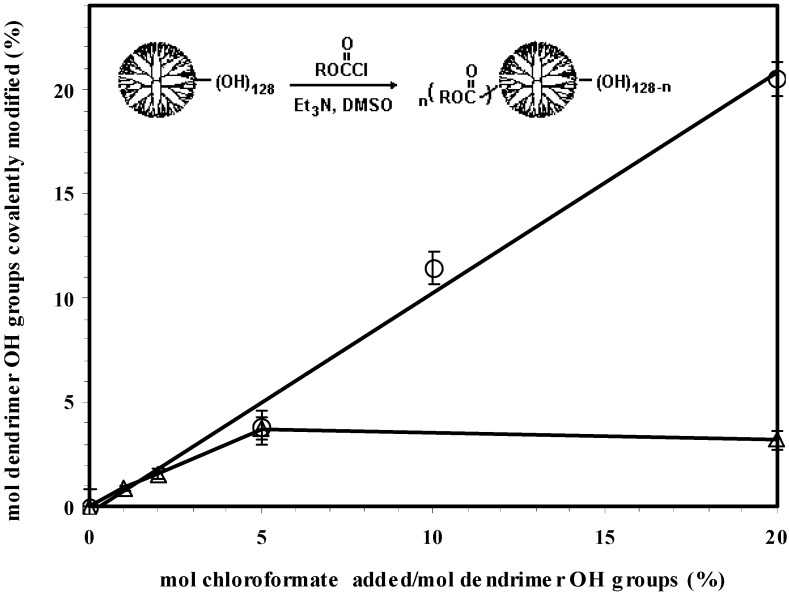
Partially hydrophobic modification of the G_5_OH dendrimer with dodecyl (Ο) or cholesteryl (Δ) chloroformate (the percentage of hydroxyl groups hydrophobized was determined by the NMR ratio of the resonance of dodecyl methyl proton at 0.83 ppm or cholesteryl = CH^6^ proton at 5.3 ppm to the corresponding dendrimer nucleus NH proton at 7.78 ppm).

In this study, a series of hydrophobic modifications were carried out by conjugating the G_5_OH dendrimer with either dodecyl chloroformate (a flexible aliphatic chain) or cholesteryl chloroformate (a rigid lipid found in abundance in biological systems). The amount of dodecyl chloroformate was adjusted to 5, 10 and 20 mole % of dendrimer hydroxyl groups, and the amount of cholesteryl chloroformate was 0.86, 2, 5, and 20 mole % of dendrimer hydroxyl groups. These molar ratios were based on the assumption that the G_5_OH dendrimer contained 128 end-terminal OH groups. Commercially available PAMAM dendrimers are known to have structural defects, reducing the number of available end groups and molecular weight. GPC analysis of the unmodified dendrimer (supporting information) gave a molecular weight of 28.5 kD and a polydispersity of 1.007, indicating that the material was of high quality. The corresponding modified dendrimers were designated as D1, D2, D3 and C1, C2, C3, C4, respectively. As shown in Table 1 and Figure 1, structural analysis by ^1^H NMR data indicated that the conjugation of the G_5_OH with dodecyl chloroformate followed a stoichiometric relationship in the investigated range. It was found that the maximum amount of cholesteryl moieties that could be introduced into the dendrimer was less than 5%. It is probable that steric hindrance caused by the bulky and rigid configuration of the cholesteryl group limited the amount of substitution. All the obtained products showed affinity to polar solvents such as dimethylsulfoxide (DMSO), dimethylformamide (DMF), and tetrahydrofuran (THF). It was found that high water solubility of the G_5_OH dendrimer was no longer observed if more than 5% of hydroxyl groups were substituted with the dodecyl groups or 2% were substituted with the cholesteryl groups. In contrast to the unmodified G_5_OH dendrimer that possesses a hydrodynamic diameter of 5.8 nm, the hydrophobized derivatives, through hydrophobic association, formed aggregates with hydrodynamic diameters ranging from 8 nm to 14 nm after sonication in physiological solutions. Their sizes and morphologies can be altered by many factors including the level of derivation, temperature, buffer, concentration, and sample preparation method. A detailed aggregation behavior of the water-soluble amphiphile C2 has been reported previously [39]. In general, the aggregates formed by cholesteryl dendrimers were larger than those formed by the dodecyl dendrimers. 

**Table 1:** The physicochemical properties of hydrophobically modified and unmodified dendrimers.

Sample ^#(a)^	% Hydrophobized groups^(b)^	Solubility in water ^(c)^	Hydrodynamic diameter ^(d)^ (nm)
G_5_OH	0	S	5.86
D1	3.8	S	8.2
D2	11.5	IS	9.6
D3	20.5	IS	10.2
C1	0.86	S	6.0
C2	1.56	S	12.2
C3	3.75	IS	13.8
C4	3.2	IS	--

^(a)^ D and C represent the dodecyl derivatized and the cholesteryl derivatized dendrimers, respectively; ^(b)^ Measured by ^1^H-NMR; ^(c)^ The solubility was tested by adding ~5 mg of each sample into 1.0 mL of solvent and shaking for 10 minutes. The sample was judged soluble (S) if a clear solution formed and insoluble (IS) if a cloudy solution formed; ^(d)^ The samples in PBS (~1 × 10^-4^), after sonicaion were meaured by DLS at 37 °C. The hydrodynamic diameter of the particles based on number was calculated from a best-fit mathematical model CONTIN. The resulting values were averaged from five replications with a mean± standard deviation of 3.0 nm.

Nourse *et al*. [40] carried out an extensive physicochemical characterization on the G_5_OH dendrimer, and reported that it had a partial specific volume slightly greater than a typical globular protein. They also reported that the G_5_OH dendrimer behaved as a discrete particle in aqueous solutions. This observation agrees with our results as indicated by the light scattering data in Table 1 for the G_5_OH unmodified dendrimer.

As dendrimers increase in generation number, there is a transition from an open dome-shaped structure at lower generations to a closed spheroid shape at higher generations [41,42,43]. This transition takes place between generations 2 and 5, so that by generation 5 the molecular structure of the higher generation dendrimers has been described as a soft spongy interior with well defined interior hydrophobic cavities surrounded by a dense outer shell, which in the case of the G_5_OH dendrimer is hydrophilic. This dense hydrophilic outer shell is largely responsible for shielding of the dendrimer interior and preventing intra-molecular interactions. 

At 34% w/w concentration, the rheological properties (storage and loss moduli) of G_5_OH dendrimers closely matched those of the total lens crystallin protein soluble fraction from porcine lenses [37]. Similarly, they matched the values of refractive index (~1.4) and density (~1.08). However, one key difference was noted in the rheological behavior of the dendrimer and the natural lens crystallins. The crystallins showed shear thinning and non-Newtonian behavior, indicating complex molecular interactions [36], while the G_5_OH dendrimers exhibited strictly Newtonian behavior [42,44]. These crystallin interactions allow for the formation of short range lattices and are considered important in both the transparency and the accommodation or focusing properties of the lens in vision. One method of overcoming these rheological differences and creating dendrimer interaction is to conjugate hydrophobic surface molecules to the dendrimers. In addition to causing particle interaction and affecting rheological properties of the dendrimers, the hydrophobic derivatives help to trap the dendrimers in an amphiphilic gel, preventing them from escaping from a prosthetic lens refilling material, thus decreasing the likelihood of dendrimers reaching the cornea or retina and causing an inflammatory response *in-vivo*. 

**Figure 2:**
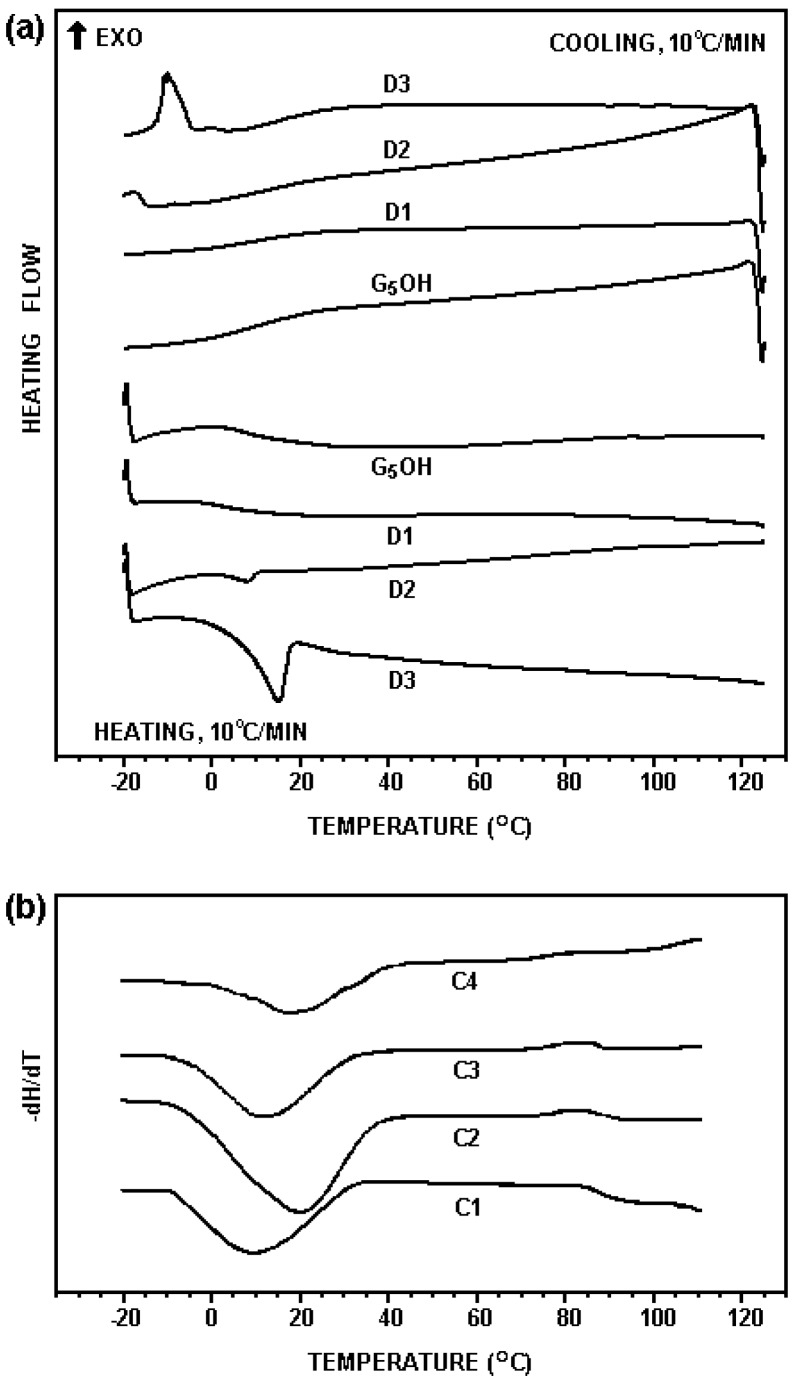
DSC profiles of hydrophobically modified and unmodified dendrimers. (a) scans of the third cooling (top) and heating (bottom) for the G_5_OH dendrimer and the dodecyl dendrimers; (b) scans of the third cooling for cholesteryl dendrimers, processed as the first derivative.

The thermal transitions of the G_5_OH dendrimer and its hydrophobic derivatives were studied by DSC. As seen in Figure 2(a), the dodecyl dendrimers with various degree of dodecyl substitution had similar glass transition temperatures (T_g_) as that of the G_5_OH dendrimer (12.3 °C). A crystallization exotherm was observed at –15.1 °C in the DSC profiles of D3, suggesting that 20% dodecyl modified dendrimer showed crystallization to some extent. None of the cholesteryl modified dendrimers showed signs of crystallization as seen in Figure 2(b). The *T_g_* of C1, C2 and C3 were 14.5, 20.3 and 16.5 °C, respectively, which were higher than that of the unmodified dendrimer. The curve of the sample C4 showed multiple transitions, implying that this sample was a mixture of the dendrimers with a wide range of different levels of cholesteryl modification. As the glass transition temperatures of all the samples tested were far below 37 °C, they behaved as liquids under the experimental condition for cell viability testing. 

**Figure 3:**
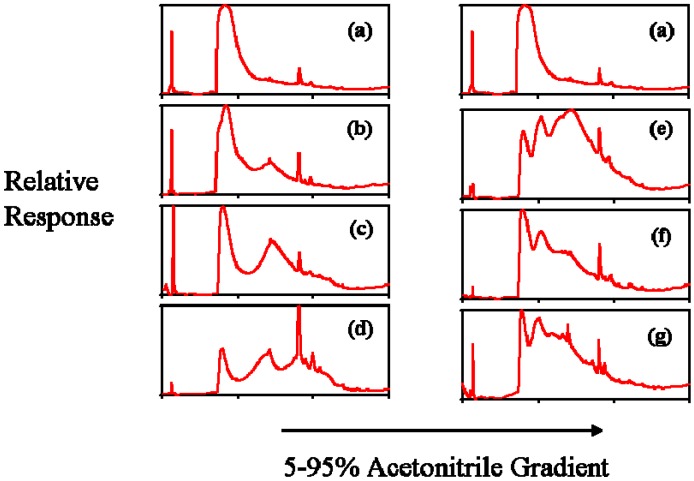
HPLC UV tracings at 230 nm of control and modified dendrimers analyzed on reverse phase C18 columns to show hydrophobicity. (a) G_5_OH unmodified dendrimer (b) C1 (c) C2 (d) C3 (e) D1 (f) D2 (g) D3.

Figure 3 shows the tracings from the reverse phase HPLC using a 5% to 95% acetonitrile gradient. With increasing hydrophobic modification, the dendrimers should require an increased organic content to be washed from the columns that would be evidenced by a shift to the right in the tracings. This shift is clearly seen in the cholesteryl derivatives. With the dodecyl derivatives, there is a clear shift between the unmodified G_5_OH dendrimer and D1, or the 5% dodecyl dendrimer. However, D2 and D3, the 10% and 20% dodecyl derivatives, appeared to come off the C18 column at a lower acetonitrile concentration than D1. This observation suggests that the dodecyl chains interacting with the column were less than those conjugated on the samples, as indicated by the NMR results, which clearly show the stoichiometric derivatization of the dodecyl groups. One possible explanation is that the dodecyl chains were partially shielded, thus preventing their interaction with the column. Monte Carlo simulations and studies of dendrimers in various solvent conditions showed that in poor solvents, back-folding of the peripheral groups can be expected [45,46]. It is possible that the flexible dodecyl hydrophobic chains could actually back-fold into the more hydrophobic interior of the dendrimer, or aggregation in part could be caused by internalization of adjacent dendrimer dodecyl chains. Encapsulation of hydrophobic guest molecules by dendrimers has been shown by many studies, which was covered in a recent review [4]. G_5_OH PAMAM dendrimers have been employed in a noncovalent drug inclusion complex study where it was demonstrated that methotrexate was internalized by dialysis in distilled water [47], indicating that these particular dendrimers are capable of this type of inclusion. This concept will be revisited when the rheology of these dendrimers is discussed. 

### 2.2. In Vitro Cell Viability

**Figure 4:**
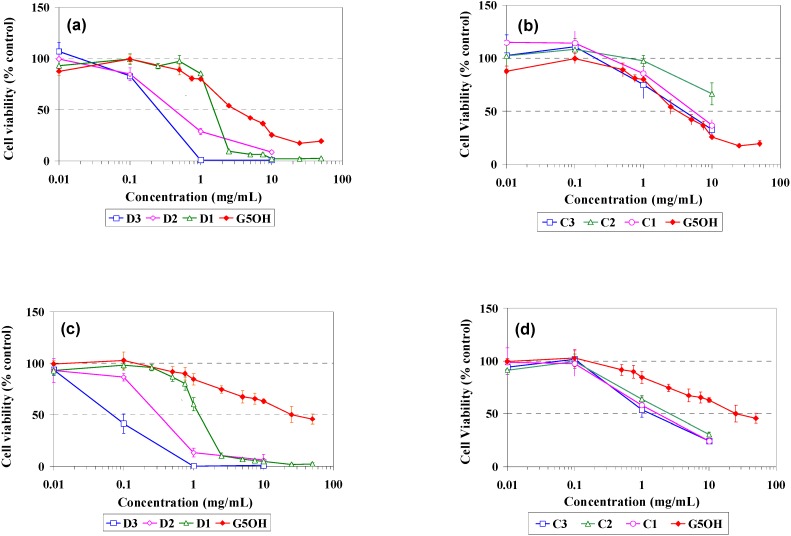
Cell viability curves of dendrimers in tissue culture cell lines: (a) Dodecyl modified dendrimers in PLE cells (b) Cholesteryl modified dendrimers in PLE cells (c) Dodecyl modified dendrimers in CHO cells (d) Cholesteryl modified dendrimers in CHO cells.

*In vitro* cell viability studies were performed on PLE cells isolated in our lab and also in CHO cells from ATCC, which is a commonly used cell line in studies of genetics, toxicity screening, gene expression and expression of recombinant proteins. The cell growth inhibition versus dendrimer concentration is shown in Figure 4 for G_5_OH unmodified and modified dendrimers. 

There are several significant differences that can be noted. First, it can be seen from Figure 4 (c), in CHO cells, the D1, D2 and D3 dendrimer IC_50_ values are 1.23, 0.37, and 0.07 mg/mL, respectively. Similar results were obtained for the PLE cells [Figure 4 (a)]. Thus, the addition of the hydrophobic chains increases toxicity in proportion to the modification as indicated by NMR as listed in Table 1. On the other hand, the cholesteryl modified dendrimers in PLE cells Figure 4 (b) and CHO cells Figure 4 (d) did not show this correlation, but are all relatively similar in their response, regardless of the amount of modification. In addition, CHO cells are fairly tolerant to the unmodified G_5_OH dendrimers having an IC_50_ of ~25 mg/mL while the PLE cells are much less tolerant having an IC_50_ of ~3 mg/mL which is almost an order of magnitude lower. The cholesteryl modified dendrimers actually showed slightly reduced toxicity when compared to the unmodified dendrimers in PLE cells, while in CHO cells the cholesteryl modified dendrimers show IC_50_’s of 1-3 mg/mL. This is comparable to but slightly less tolerant than the values obtained from the PLE cells, but well below the value of 25mg/mL obtained from the unmodified G_5_OH dendrimers in CHO cells. 

**Figure 5:**
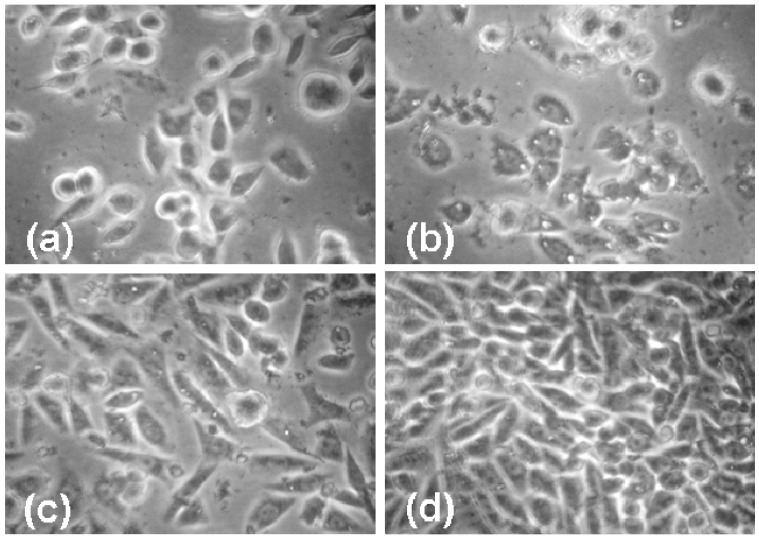
Phase contrast images showing morphology and density effects of dendrimers at 10 mg/mL on CHO cells after 70 hours incubation. (a) 2% Cholesteryl modified dendrimer (C2) (b) 5% Dodecyl modified dendrimer (D1) (c) G_5_OH unmodified dendrimer (d) Control, no additions.

Figure 5 shows the cell morphology of CHO cells exposed to 10 mg/mL dendrimer for 70 hours. While the C2 2% cholesteryl dendrimers do inhibit cell growth, there are very few vacuoles present, and the cells look relatively normal. The cells exposed to the D1 5% dodecyl dendrimer appear highly stressed with many vacuoles. The CHO cells exposed to the unmodified G_5_OH dendrimer show less growth inhibition than cells exposed to modified dendrimers. The cells show more vacuoles than those exposed to the cholesteryl modified dendrimers but significantly less than those exposed to the dodecyl modified dendrimers. 

**Figure 6:**
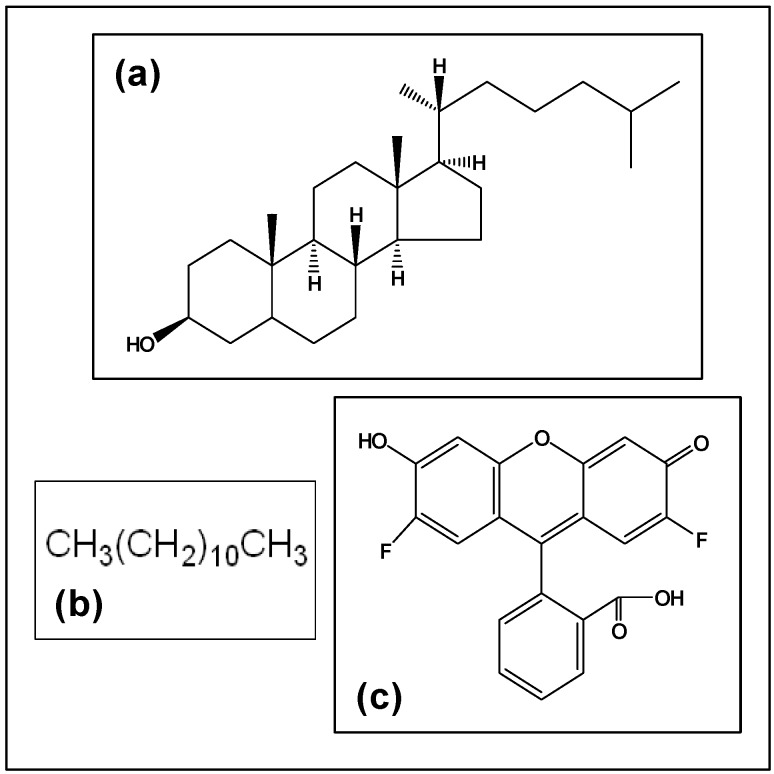
Structures of compounds. (a) Cholesterol (b) Dodecane (c) Oregon Green 488 [24].

Neutrally charged hydrophilic macromolecules are known to cross through biological barriers (cell membrane, endosomal membrane, microvessel wall, etc) depending on molecular weight or size, and geometry or architecture. However, there is ample evidence that the hydrophobic modification of dendrimers can radically affect their behavior in transport. Figure 6 gives the structure of cholesterol and dodecane, the two hydrophobic groups used in this study. Also given as a comparison is the structure of Oregon green 488, a fluorescent compound, which was used to modify G_5_NH_2_ PAMAM dendrimers in another group’s study [31]. Yoo *et al.* used the modified dendrimers to transport oligonucleotides into the nuclei of HeLa cells. The results showed that the abilities as a delivery agent for the oligonucleotides were greatly enhanced (~5 fold) by the presence of the hydrophobic fluorescent label even at a 1.0:0.8 mole ratio of Oregon green 488:dendrimer. Measurements were also made confirming the dendrimers’ presence in the nuclear fractions of the cells. It was suggested that the hydrophobic fluor moieties enhance the ability of the dendrimer to disrupt endosomal membranes and thus traffic to the cytosol and nucleus. 

Applying these observations to our study, we postulate the following, even though we have not employed an ionic dendrimer. The addition of hydrophobic elements to the surface of the dendrimers would be expected to enhance the binding of the modified dendrimer to membrane components and subsequent entrance into the cells, affecting the biocompatibility. Cholesterol is a rigid lipid that is found in abundance in cell membranes and is involved in cell metabolism. It is a molecule similar in size and hydrophobicity to the Oregon green 488 dye. Dodecane is a non-rigid hydrophobic chain. It is expected that the response to these materials would be quite different. When examining the results from the toxicities of these hydrophobized dendrimers toward the CHO and PLE cells, the most notable difference is that in the case of the dodecyl modification, there is a direct correlation between the increase in hydrophobic surface conjugation and toxicity. There was no direct relationship between toxicity of the cholesteryl dendrimers and amount of surface conjugation with cholesteryl units. With the cholesterol series, there was an initial increase in toxicity of the 1% modified dendrimer as seen in CHO cells. There was no significant difference between the 1% and 4.6% cholesterol dendrimers. In PLE cells, the cholesterol modified dendrimers were slightly less toxic than the unmodified dendrimer. Since the conditions of our MTT viability assay were comparable to those performed by Malik [19], it is reasonable to compare our results with theirs obtained using B16F10 cells. They showed IC_50_ values for G_4_ amine terminated dendrimers to be in the range of 0.01 to 0.1 mg/mL, while with the anionic carboxyl terminated G_3.5_ or G_7.5_ dendrimers the cells did not reached the level of IC_50_ toxicity at 2 mg/mL. Given these comparisons, the unmodified OH terminated dendrimers in CHO cells have similar biocompatibility to the carboxyl terminated dendrimers in biocompatibility, with an IC_50_ of 25 mg/mL, while the highly substituted D3 dendrimers showed a similar toxicity to the amine terminated dendrimers (IC_50_=0.07 mg/mL). It is safe to infer that the cholesterol is benign to the cells. So, evidence suggests that the toxicity of the dodecane moiety itself contributes to the reduced biocompatibility with increased concentration, though we believe that the amphiphilic character of the dendrimers increases their uptake into the cells. The difference in response between CHO and PLE cells to the G_5_OH dendrimers is not clear, and will take further study to ascertain.

### 2.3. Dendrimer Rheology

We have previously demonstrated the possibilities of making lens refilling materials using a two component system, combining a polymer gel with nanoparticles [34]. The viscoelastic properties of lens crystallins and G_5_OH dendrimers has also been reported by our group [37]. One main advantage to using amphiphilic materials to form the desired nanocomposites is that the hydrophobic attractions between molecules should greatly reduce the possibility of the dendrimers escaping from the nanocomposite lens material and causing damage to other ocular tissues. 

**Figure 7:**
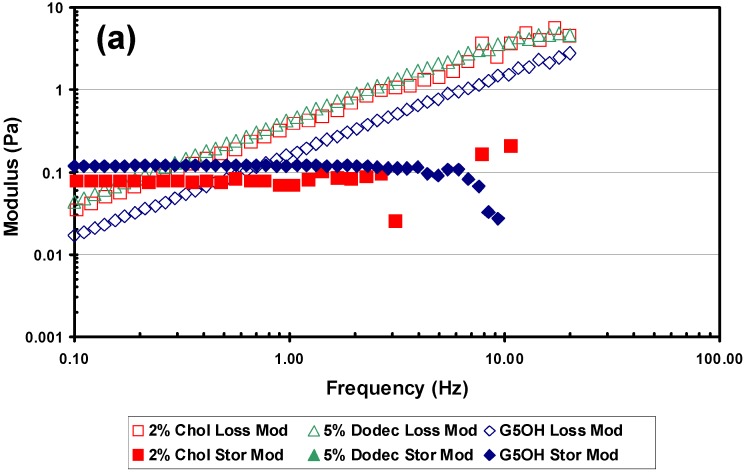

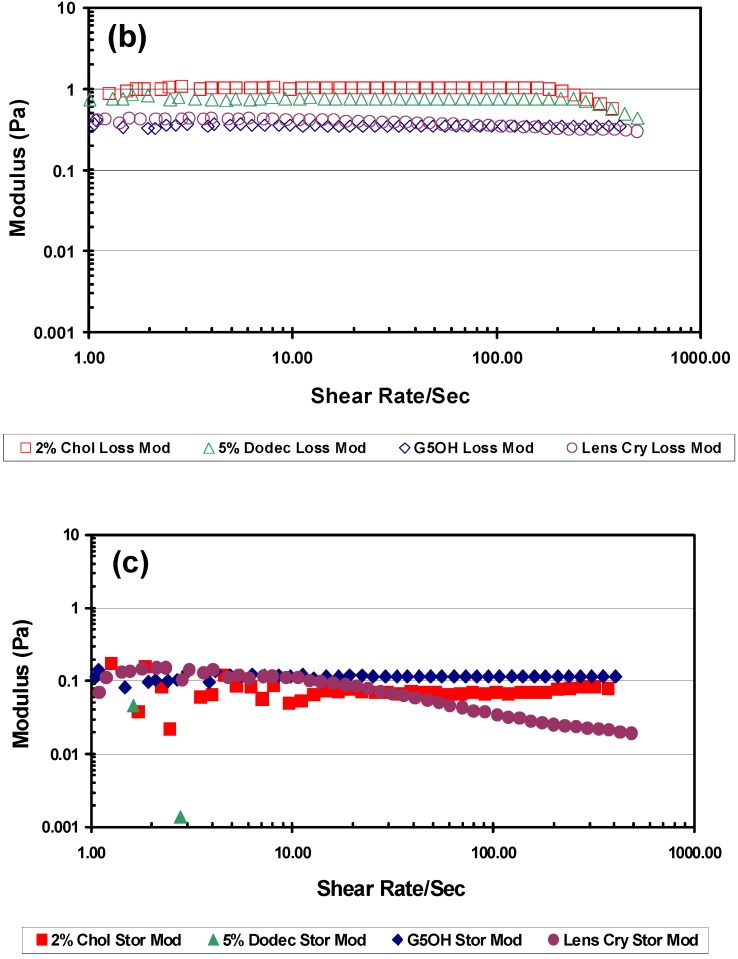
Rheological results from the Vilastic capillary rheometer. All experiments were performed at 37 °C. Dendrimers were tested at 34% w/w in PBS and lens crystallins were at physiological concentrations (~34% w/w) (a) Frequency scan at a shear rate of 20 s^-1^ comparing loss modulus and storage modulus of 2% cholesteryl (C2), 5% dodecyl (D1) modified and G_5_OH unmodified dendrimers. (b) Shear rate scan at a frequency of 2Hz, comparing the loss modulus of C2, D1 modified and G_5_OH unmodified dendrimers with porcine lens crystallins (c) Shear rate scan at a frequency of 2Hz, comparing the storage modulus of C2, D1 modified and G_5_OH unmodified dendrimers with porcine lens crystallins.

Experiments were performed to evaluate the effects of the hydrophobic modification on viscoelastic properties of the modified dendrimers. Figure 7(a) shows a frequency scan at a shear rate of 20 s^-1^, which is within the linear viscoelastic response region of the material. The hydrophobic dendrimers showed an increased loss modulus over the entire frequency range, compared with the unmodified dendrimers. This is reasonable considering the increased particle size due to aggregation of the modified dendrimers. 

Figure 7(b) and (c) show that under similar conditions, the G_5_OH dendrimer at 34% w/w in addition to matching the porcine lens crystallins in refractive index and density also has a comparable storage and loss modulus. When comparing the storage modulus of the two modified dendrimers, there is one observation that stands out. The storage modulus of the 5% dodecyl dendrimer was below the sensitivity level of the instrument and could not be measured, indicating purely liquid-like behavior. This was true for all conditions of shear and frequency values tested. The storage modulus values of the 2% cholesteryl dendrimer were similar to but slightly lower than the unmodified dendrimer. If the hydrophobic dodecyl groups remain on the exterior shell of the dendrimers, the hydrophobic interaction of these groups between dendrimers should create a measurable storage modulus. The fact that we could not measure the storage modulus supports the hypothesis that the dodecyl groups could be internalized into the core of the dendrimers. 

The rheology of starburst PAMAM dendrimers, was investigated by Uppuluri *et al*. The results revealed that PAMAM dendrimers exhibited Newtonian flows in ethylenediamine solutions, and non-Newtonian viscoelastic response in bulk [42,43]. 

### 2.4. Dendrimers in Ocular Applications

Dendrimers have been used in a number of ocular applications. These applications have been reviewed by Cheng *et al*. [16]. We will mention several pertinent applications. For use in the anterior segment of the eye, Vandamme [17] studied the use of amine, carboxylate and hydroxyl surface groups in several series of poly(amidoamine) (PAMAM) dendrimers (generations 1.5 and 2-3.5 and 4) in buffered phosphate solutions for controlled ocular drug delivery in rabbits. The duration of residence time was evaluated after solubilization of series of PAMAM dendrimers with 2 parts per thousand (w/v) of fluorescein. The New Zealand albino rabbit was used as an *in vivo* model for qualitative and quantitative assessment of ocular tolerance and retention time after a single application of 25 μL of dendrimer solution to the eye. The same model was also used to determine the prolonged miotic or mydriatic activities of dendrimer solutions, some containing pilocarpine nitrate and some tropicamide, respectively. The dendrimers induced slower release of the drugs as a result of encapsulation, and exhibited some bioadhesive properties. Physiochemical parameters of the prepared eye drops suggested that the dendrimer solutions would not cause any ocular irritations. Surprisingly the amine terminated dendrimers did not appear to induce any more irritation that the hydroxyl and carboxyl terminated dendrimers. 

For use in the posterior segment of the eye, Marano *et al*. [18] have synthesized polycationic lipophilic peptide core dendrimers. These agents were used to transfect human retinal pigment epithelium cells with an oligonucleotide called ODN1. This in turn was reported to cause a reduction in the production of the protein vascular endothelial growth factor. This could be used in the control of neovascularization which when out of control, can cause blindness.

Several groups are working on corneal tissue engineering materials involving dendrimers: Duan *et al*. [13] used G_2_ polypropyleneimine octaamine dendrimers to generate highly crosslinked collagen with mechanical properties that would make it appropriate for use as a corneal tissue-engineering scaffold. Using carbodiimide 1-ethyl-3-(3-dimethyl aminopropyl) carbodiimide hydrochloride (EDC), the multifunctional amine terminated dendrimers were introduced as novel multifunctional cross-linkers, after the activation of the carboxylic acid groups of glutamic and aspartic acid residues in collagen. Young's modulus of the dendrimer cross-linked gels was in the range of 1-5 MPa. Optical transparency of the dendrimer cross-linked collagen was fairly good. Glucose permeation results demonstrated that the dendrimer cross-linked collagen had higher glucose permeability than natural human cornea. Dendrimer cross-linked collagen gels supported human corneal epithelial cell growth and adhesion, with no cell toxicity. The dendrimer cross-linked collagen gels showed promise for scaffolds for corneal tissue engineering.

Grinstaff [19] has done work on designing and evaluating corneal adhesives prepared from “biodendrimers” using a photocrosslinking reaction and also a peptide ligation reaction to couple individual dendrimers together to form an adhesive. For example, they have synthesized a G4 poly(glycerol-succinic acid) dendrimer terminated with OH groups. Subsequent cross-linking of their dendrimers gives hydrogel adhesives. These adhesives were successfully used to repair corneal perforations; close the flaps produced in LASIK procedures, and secure corneal transplants.

The dendrimers for drug delivery do not directly apply to our application. However, these examples give indications that the above dendrimers can be used in ocular applications with minimal detrimental effects to the ocular tissues. The dendrimers cited for tissue engineering also are being used differently from the applications of the dendrimers set forth in this study in that they are employed as covalent cross-links to help form hydrogels in the MPa range. We are interested in much softer hydrogels in the 1 KPa range. It is not our intent that the hydrophobically modified dendrimers will be covalently incorporated into a hydrogel network, but rather will be kept by hydrophobic interactions in a hydrogel network formed by hydrophobically modified polymers that may not be cross-linked, or may be cross-linked at a low density. If we find that the hydrophobic interactions are insufficient to keep the nanocomposite intact, a minimum number of covalent bonds may need to be added. 

## 3. Experimental Section

### 3.1. Materialsand Methods

Fifth generation hydroxyl-terminated poly(amidoamine) dendrimers (G_5_OH, molecular weight 28.4 kD, approximately 128 hydroxyl groups) were obtained from Dendritech, Inc. (Midland, MI, USA). Cholesteryl chloroformate (97%), dodecyl chloroformate (98%), triethylamine (Et_3_N) (97%), dimethyl sulfoxide (DMSO) (HPLC grade), all tissue culture regents 3-[4,5-methylthiazol-2-yl]-2,5-diphenyltetrazolium bromide (MTT), minimum essential medium (MEM), calf serum, and antibiotics were obtained from Sigma-Aldrich (St. Louis, MO, USA), and used as received. CHO cells were purchased from American Type Culture Collection (Manassas, VA, USA), and PLE cells were harvested from fresh pig lenses as described by others [48]. 

^1^H-NMR spectra were recorded on a Varian Inova-600 MHz spectrometer in DMSO-*d_6_* (Aldrich). VNMR software was used for integration values for structural analysis. Dynamic light scattering (DLS) measurements were obtained with a Brookhaven Instrument **(**Holtsville, NY, USA**)** equipped with a laser (514-nm incident wavelength) and BI9000AT correlator at a 90-degree angle. Differential scanning calorimetry (DSC) was performed on a DSC-4 **(**Perkin Elmer Waltham, MA, USA**).** Approximately 5 mg of sample was loaded in an aluminum pan for measuring. The heating-cooling cycle was performed three times, and the data was collected from the third cycle. Glass-transition temperatures (T_g_) were determined as the midpoint of the inflection tangent during the third cooling scan and crystallization temperatures (T_c_) were the onset point of the crystallization exotherm in the third cooling scan. 

Reverse phase high performance liquid chromatography (HPLC) was performed on a C_18_ reverse-phase column (Phenomenex, 4.6 × 150 mm^2^) using water/acetonitrile as the mobile phase (5%-95% gradient over 90 minutes, 1% every minute with a lag time of 5 minutes). Samples size was 50 μL at 1 mg/mL and the flow rate was 0.5 ml/min. The UV detector wavelength was set at 230 nm for all the samples**.**

### 3.2. Partially Hydrophobized G_5_OH Dendrimer

The cholesteryl-G_5_OH dendrimers were synthesized as described previously [38,39]. A similar procedure was used to prepare the partially modified G_5_OH dendrimers with dodecyl chains. Briefly, solvent-free G_5_OH (1.0 g) was dissolved in anhydrous DMSO (20 mL). Dodecyl chloroformate and Et_3_N were then added sequentially. The starting molar ratio of chloroformate to the number of dendrimer hydroxyl groups was varied from 5%-20%. Ten percent excess of Et_3_N was used, based on the amount of chloroformate in the reaction. The reaction was carried out for 24 hours with stirring under nitrogen at room temperature. The reaction mixture was then dialyzed against ethanol/water mixtures starting with 1:1 ratio and then dialyzed extensively against deionized water (MWCO 6-8 kD). Cholesterol dendrimers were further extracted with diethyl ether to remove unreacted cholesterol. The resulting aqueous solution was lyophilized, and the obtained product was stored at −20 °C.

*The dodecyl derivatized dendrimers:* ^1^H-NMR (ppm): δ_dode_ ~0.8 (C*H*_3_) 1.2-1.5 ((C*H*_2_)_11_); δ_G5OH_: 2.18 (CH_2_C*H*_2_CONH), 2.41 (NHCH_2_C*H*_2_N=), 2.62 (COCH_2_C*H*_2_N=), 3.08-3.09 (NHC*H*_2_CH_2_OH, NHC*H*_2_CH_2_N=), 3.38 (NHCH_2_C*H*_2_OH), 7.79 (N*H*CH_2_CH_2_N=), 7.92 (N*H*CH_2_CH_2_OH) 

*The cholesteryl derivatized dendrimers:* ^1^H-NMR (ppm): δ_chol_: 0.64 (C*H*^18^_3_), 0.83-0.84 (C*H* ^26, 27^_3_)_2_), 0.97 (C*H*^19^_3_), 1.10 (C*H*^21^_3_) 4.30-4.35 (-C*H*^3^-OCO), 5.24-5.31 (=C*H*^6^-); δ_G5OH_: 2.18 (CH_2_C*H*_2_CONH), 2.41 (NHCH_2_C*H*_2_N=), 2.62 (COCH_2_C*H*_2_N=), 3.08-3.09 (NHC*H*_2_CH_2_OH, NHC*H*_2_CH_2_N=), 3.38 (NHCH_2_C*H*_2_OH), 7.79 (N*H*CH_2_CH_2_N=), 7.92 (N*H*CH_2_CH_2_OH) 

### 3.3. In Vitro Cytotoxicity Testing

PLE cells were obtained from porcine eyes using a modified procedure described by others [48]. Briefly, porcine eyes were dissected under sterile conditions within 2 hours of sacrifice. The cornea was excised and the anterior capsule was punched with an 8.00 mm trephine and gently removed with forceps. The contents of the capsular bag were easily prolapsed, and the posterior portion of the lens capsule was left attached to the sclera by the ciliary body and zonules. The remainder of the sclera, below the plane of the posterior lens, and vitreous were cut away. The attached lens capsule was then submersed in minimal Eagle medium (MEM) containing 10% fetal calf serum (FCS) with antibiotics (gentamycin, penicillin, streptomycin, and amphotericin B) to allow attached lens epithelial cells to multiply. This media was used for all of the subsequent experiments and was changed every three days until the cells were confluent on the posterior capsule. Cells were then trypsinized, and the resulting detached cells were collected and transferred to tissue culture flasks. The cells were grown and used in our experiments.

The tested dendrimers were purified by dialysis (MWCO 6-8 kD) against fifty volumes and four changes of deionized water and then lyophilized. Testing was carried out by following the procedure for the MTT assay as outlined by Malik *et al.* [26]. Testing was performed twice to ensure that the results were reproducible. The MTT assay is based on the measurement of the mitochondrial reductase activity, an enzyme active only in living cells which converts the yellow soluble MTT to that of purple water-insoluble formazan crystals. CHO and PLE cells were seeded in 96 well plates, 5,000 cells/well, in MEM (0.15 mL) containing 10% fetal calf serum with antibiotics and allowed to adhere for 24 hours at 37 °C. Fresh media containing dendrimers at 0.01 to 50 mg/mL was then added to the cells. After 70 hours of incubation, MTT (20 μL, 5 mg/mL) dissolved in medium was added and cells were incubated an additional 5 hours. Media was then removed and DMSO (100 μL) was used to dissolve the formed formazan crystals. Optical density at 550 nm was measured in a 96 well plate reader (BioRad Hercules, CA). Using the optical density readings, the viability of cells exposed to test samples was expressed as a percentage of viability compared to control cells grown in medium without exposure to dendrimers. Using the dose response graph, the reading at which the curve crossed the 50% mark is referred to as the IC_50_ or the point at which the optical density reading is 50% of the control absorbance. This point is used as a relative measure of toxicity. Images of the cells were taken at various conditions after 70 hours of incubation, before addition of MTT. Media was removed briefly for photography.

### 3.4. Rheological Measurements

The dendrimer solutions were prepared by rehydrating the lyophilized samples at 34% w/w giving a refractive index value of 1.4 and a density of ~1.08 g/mL in Dulbecco’s Modified Phosphate Buffered Saline without Ca^2+^ or Mg^2+^ (DPBS). Porcine lens crystallins were prepared as previously reported [37].

Rheological experiments were performed with the Vilastic-3 Viscoelasticity Analyzer (Vilastic Scientific, Inc. Austin, TX). The instrument employs an oscillatory capillary tube system. Protocols were set up to perform shear rate scans at constant frequency or frequency scans at constant shear rate. The flow, volume, and pressure were measured throughout the cycle and from these, the viscous and elastic components of the shear stress, shear rate, and shear strain at the capillary tube wall were calculated by the Vilastic software. Different diameter tubes were employed to ensure testing was within the linear viscoelastic range. The system is hooked to a circulating water bath and temperature was maintained at 37 °C. 

## 4. Conclusions 

Dendrimers have unique properties among synthetic polymers. Dendrimers were selected for application because they are globular, monodisperse and have definite molecular weight, shape and size. These characteristics enable us to mimic globular lens crystallin proteins, matching some important parameters such as storage and loss modulus, density and refractive index with reproducible results. Starting with G_5_OH dendrimer, we successfully modified the molecule with hydrophobic derivatives. We will employ these materials to supply refractive index and appropriate viscoelastic properties to amphiphilic nanocomposites in accommodative lens materials. The results show that the 1%-2% cholesteryl and 5% dodecyl dendrimers are promising candidates for our applications, while the more highly modified dendrimers are not as they became water insoluble and lost their transparency. 

It was noted that the unmodified G_5_OH dendrimers were well tolerated by CHO cells. With the dodecyl modified dendrimers, there was a direct correlation between the increase in hydrophobic surface conjugation and toxicity. This was not the case with the cholesteryl modified dendrimers where no correlation was seen between conjugation and toxicity. These dendrimers are needed at a high concentration in the lens to give the required optical properties. However, it is desirable to inhibit the growth of the lens epithelial cells, so as to prevent posterior capsular opacification, or secondary cataract [34]. The critical issue will be the prevention of toxicity to the other tissue components of the eye by ensuring that there is very little loss of the nanocomposite from the lens capsule and that the materials uses are relatively biocompatible. 

The hydrophobic modification resulted in aggregation of the dendrimers in aqueous solutions due to hydrophobic interactions. An increased viscosity or loss modulus of the modified dendrimers was observed based on the rheology tests of the 2% cholesteryl and the 5% dodecyl dendrimers. Based on the reverse phase HPLC observation and rheological evaluation of the dodecyl dendrimers, it is proposed that the dodecyl chains were internalized in the dendrimers. 

Research is currently under way to incorporate these modified dendrimers in nanocomposites for lens implant materials. 

## Supporting Information

Supporting information can be downloaded online at http://www.mdpi.com/1996-1944/2/3/883/s1.
