# Supplementary material for: Surface Hydrophobic Modification of Fifth-Generation Hydroxyl-Terminated Poly(amidoamine) Dendrimers and Its Effect on Biocompatibility and Rheology
Source: Materials (Basel). 2009 Aug 4;2(3):883–902. doi: 10.3390/ma2030883 (PMC5445743; doi:10.3390/ma2030883)
Supplement: Supplementary file 1 [file materials-02-00883-s001.pdf]

Supporting Information

Surface Hydrophobic Modification of Fifth-generation Hydroxyl-terminated Poly(amidoamine) Dendrimers and Its Effect on Biocompatibility and Rheology

Paul D. Hamilton, Donghui Z. Jacobs, Brain Rapp and Nathan Ravi *

^{1}H NMR spectra to support data for Table 1 and Figure 1: For synthesis of hydrophobically modified dendrimers, dodecyl chloroformate was adjusted to 5, 10 and 20 mol % of G_5OH PAMAM dendrimer hydroxyl groups, and the amount of cholesteryl chloroformate was 0.86, 2, 5, and 20 mol % of dendrimer hydroxyl groups. The corresponding modified dendrimers were designated as **D1, D2, D3** and **C1, C2, C3, C4,** respectively. ^{1}H NMR spectra were recorded on a Varian Inova-600MHz spectrometer in DMSO-d_6 (Aldrich). VNMR software was used for integration values for structural analysis.

Figure 1. ^{1}H NMR ratio of the resonance of dodecyl methyl proton at 0.83 ppm to the corresponding dendrimer nucleus NH proton at 7.78 ppm.

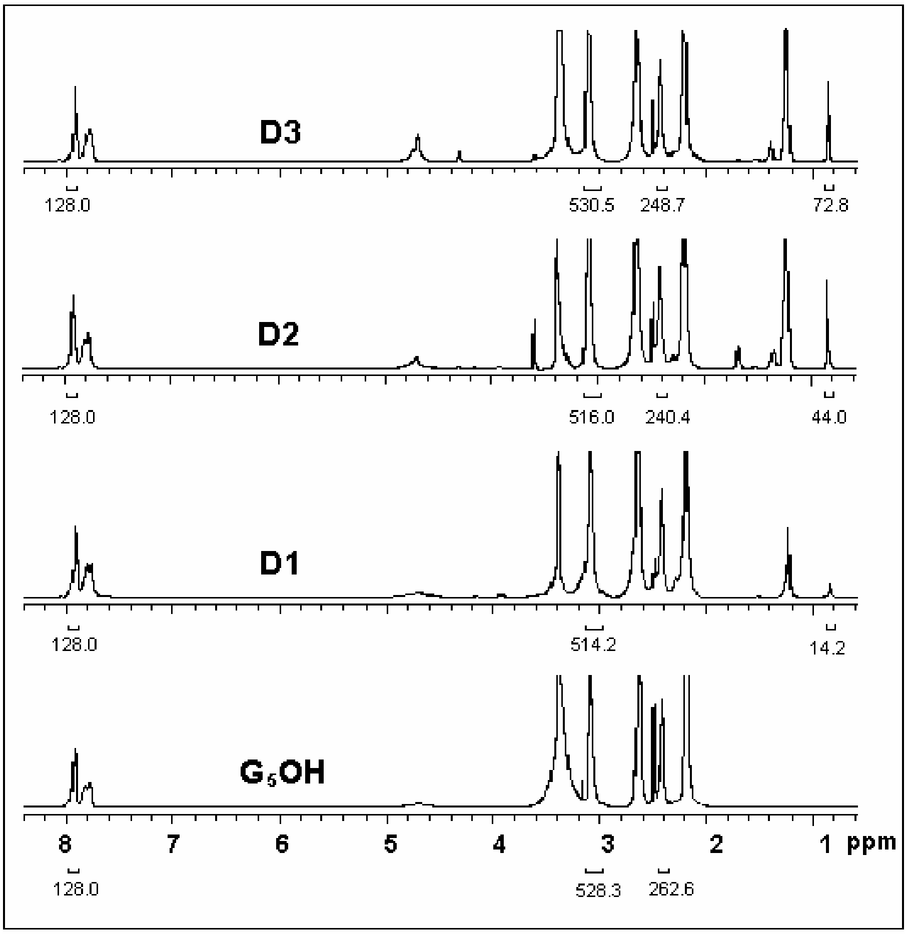

Figure 2. ^{1}H NMR ratio of the resonance of the cholesteryl =CH^6 proton at 5.3 ppm to the corresponding dendrimer nucleus NH proton at 7.78 ppm.

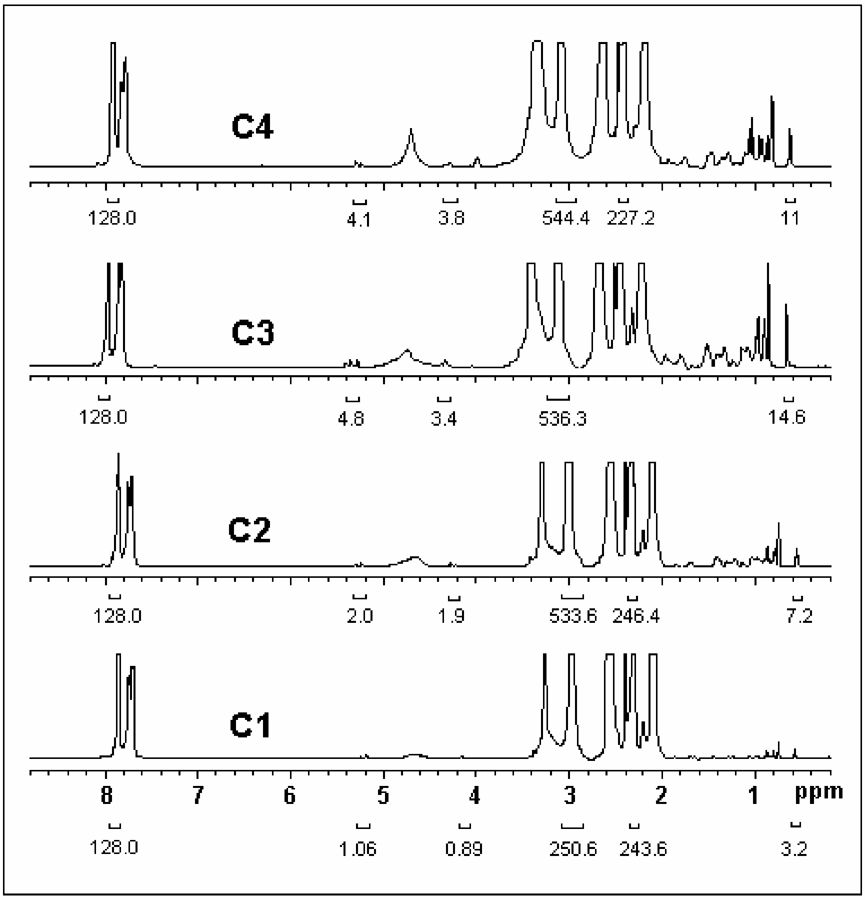

Gel Permeation Chromatography Data (GPC)

Lens Crystallins

Preparation of Unfractionated Crystallins for Rheology Experiments: All of our experiments utilized lenses obtained from six month old juvenile pigs through a local abattoir. The lenses were homogenized and spun in a Beckmann model 80 ultracentrifuge (Beckman Coulter Instruments, Fullerton, CA) using a SW41 rotor at 30,000 rpm for 60 minutes (~100,000 x g). Water soluble unfractionated crystallins were at the bottom of this centrifuged sample, while a less dense water insoluble cell fraction formed a layer on top of the crystallins. These unfractionated crystallins were acquired by pipetting them out from underneath the water insoluble cell fraction. An Abbe refractometer was used to measure the refractive index (RI) of the unfractionated crystallins with a visible light wavelength of 552 nm at 37 °C (ATAGO Abbe Refractometer NAR-1T, Kirkland, WA). The lens crystallins had a refractive index of ~1.395 and a density of 1.09 g/mL.

The water soluble unfractionated porcine lens crystallins are made up of a complex mixture of globular proteins with a large range of molecular weights from >10^6 Daltons to ~22,000 Daltons, classified into α, β, & γ crystallin families depending upon their molecular weight, structure, subunit configuration and material properties. (Berman, E. R. *Biochemistry of the Eye*. Plenum Press, New York. 1991).

Figure 3. UV chromatograms of the porcine lens crystallin protein molecular weight (MW) distribution. GPC was performed on two TSK 4000A columns (Tosoh Bioscience, Montgomeryville, PA) in series connected to a UV Waters 490E detector set at 280 nm (Waters Corporation, Milford, MA). Viscotek (Houston, TX) TriSEC 3.0 software was used to acquire data and process it for graphing.

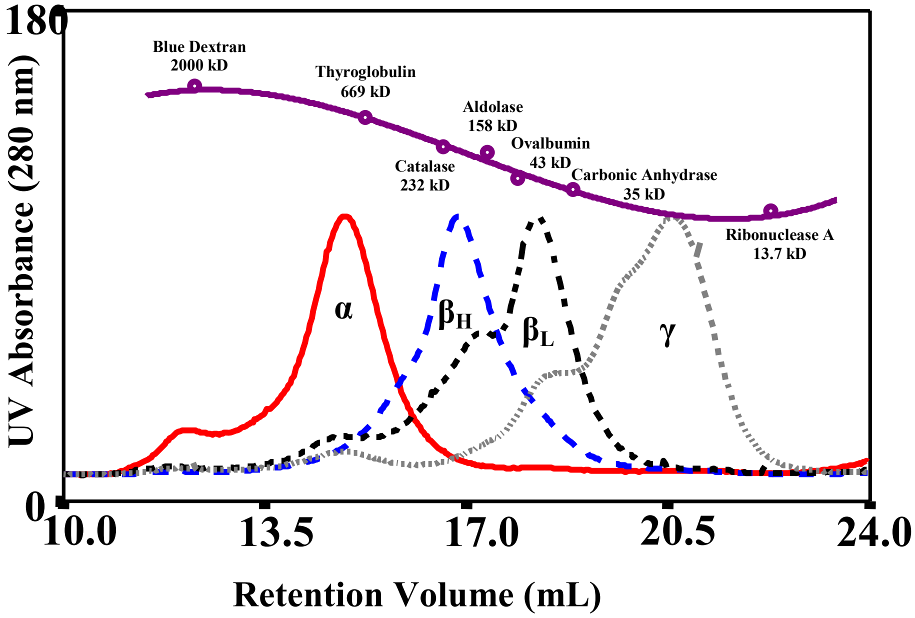

GPC Data for G_5OH Dendrimer and One Example of GPC for a Modified Dendrimer

GPC was performed with a single TSK 4000A column was used coupled to a Viscotek on-line Triple Detector 300TDA system which included a differential refractometer (RI), a four capillary differential viscometer (VISC) and a right angle laser light scattering detector (LS). TriSEC 3.0 software was used to acquire data and process it for graphing. A PBS solution (pH=7.2) was used as eluent at a flow rate of 0.8 mL/min at room temperature. 100-150 µL of dendrimer solutions (20 mg/mL, in PBS) were injected and passed through a 0.2 µm filter.

Figure 4. Triple detector chromatogram showing the RI, intrinsic viscosity and light scattering curves of the G_5OH dendrimer.

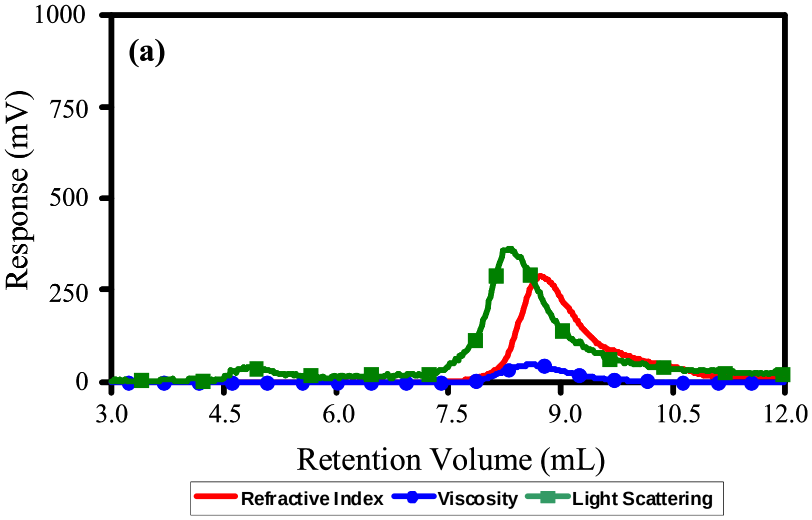

The column was standardized using polyethylene oxide standards from Viscotec. The hydroxyl dendrimer exhibited a MW of 28.5 kD and a polydispersity (Pd) of 1.007. Size calculations showed a radius of gyration of 3.6 nm, which is comparable with published values. (Uppuluri, S.; Keinath, S. E.; Tomalia, D. A.; Dvornic, P. R. Rheology of Dendrimers. I. Newtonian Flow Behavior of Medium and Highly Concentrated Solutions of Polyamidoamine (PAMAM) Dendrimers in Ethylenediamine (EDA) Solvent. *Macromolecules* **1998**, *31,* 4498-4510).

Figure 5. Triple detector chromatogram showing the RI, intrinsic viscosity and light scattering curves of a run with the G_5OH **C2** dendrimer. Note the significant increase in light scattering.

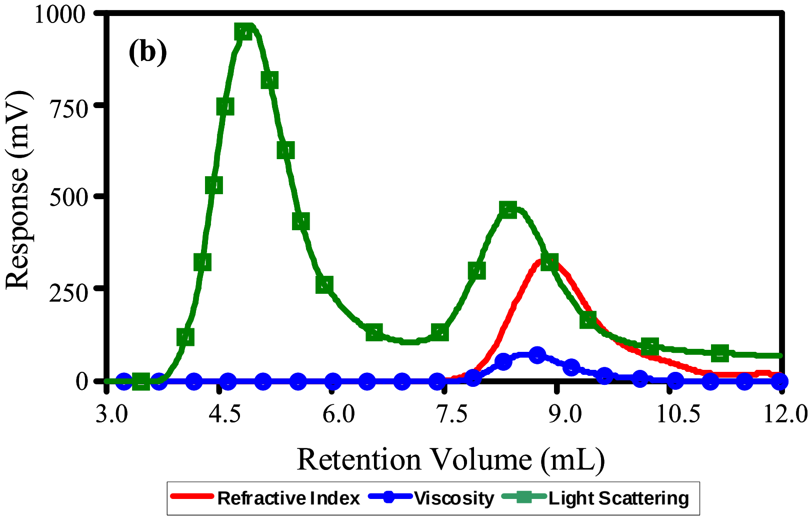

Results for GPC analysis of the hydrophobically modified dendrimers varied significantly depending on whether the dendrimers were lyophilized, sonicated, and varied depending on the ionic strength of the buffer, etc. Data obtained were consistent with the dynamic light scattering data in Table 1. Due to aggregation, size of individual dendrimer molecules was difficult to attain. See Reference: (Zhang, D.H.; Hamilton, P.D.; Kao, J.L.F.; Venkataraman, S.; Wooley, K.L.; Ravi, N. Formation of Nanogel Aggregates by an Amphiphilic Cholesteryl-Poly(Amidoamine) Dendrimer in Aqueous Media. *J. Polym. Sci., Part A: Polym. Chem.* **2007**, *45,* 2569-2575).
